# Egg Quality from Nera Atriana, a Local Poultry Breed of the Abruzzo Region (Italy), and ISA Brown Hens Reared under Free Range Conditions

**DOI:** 10.3390/ani11020257

**Published:** 2021-01-20

**Authors:** Andrea Ianni, Dayana Bartolini, Francesca Bennato, Giuseppe Martino

**Affiliations:** Faculty of Bioscience and Technology for Food, Agriculture and Environment, University of Teramo, 64100 Teramo (TE), Italy; aianni@unite.it (A.I.); dbartolini@unite.it (D.B.); fbennato@unite.it (F.B.)

**Keywords:** indigenous hen, Nera Atriana hen, biodiversity, egg quality, yolk protein profile, cholesterol

## Abstract

**Simple Summary:**

The safeguarding of genetic resources at risk of extinction represents a current topic of considerable interest. In the zootechnical field, one of the strategies that can be applied in order to pursue this scope, can be to highlight the qualitative aspects of the animal product that can attract the attention of both consumers and breeders. This study represents the first attempt to characterize the main physicochemical aspects of the eggs obtained from the Nera Atriana hen, an indigenous Italian laying hen historically bred in restricted areas of the Abruzzo region. In comparison with commercial hybrid hens, Nera Atriana hen eggs were characterized by valuable features mainly associated to the egg structure, fatty acids composition, cholesterol content, and yolk protein profile. This study should therefore be understood as a preliminary approach aimed at ensuring that interest can develop in preserving a genetic resource currently reduced to a few dozen specimens.

**Abstract:**

The selection and diffusion in the poultry sector of hybrid breeds able to produce higher amount of meat and eggs, led over time to the erosion of genetic resources. One of the strategies that can be applied in order to stem such phenomenon, concerns the valorization of the animal products, meat or eggs, obtained from indigenous poultry breeds. Therefore, the aim of this study is to characterize the qualitative aspects of eggs obtained from the Nera Atriana hen, an Italian indigenous laying hen characteristic of the Abruzzo region, making a direct comparison with a commercial hybrid reared in the same environment and applying the same feeding protocol. The trial was conducted for a period of 5 months (from March to July 2019), in which 6 egg samplings were performed (one at the beginning and additional 5 on a monthly basis); each sampling involved the collection of 15 eggs per group of animals for a total of 90 eggs per genotype. Eggs were specifically subjected to evaluations of the physical parameters, including the yolk color, and analyses aimed at determining the content of total lipids, cholesterol, and β-carotene. In addition to this, the fatty acids profile and the electrophoretic pattern of the globular proteins of the yolk were characterized. Eggs from hybrid hens were characterized by higher total weight with significantly higher values specifically associated to albumen and yolk weight and to the albumen percentage. In the eggs obtained from the local hen, higher relative percentages were instead found in the quantity of shell and yolk; furthermore, in the same samples was observed a higher yolk redness, a cholesterol concentration tending to be lower although not significant (*p* = 0.0521), and a greater amount of saturated fatty acids which could justify a lower susceptibility of the food to oxidative processes following transformation with heat treatments. With regard to the fatty acid composition, differences were specifically observed for palmitic (C16:0) and palmitoleic (C16:1 cis-9) acids that showed higher relative percentages in eggs from local breed, and for the oleic acid (C18:1 cis-9) which was more represented in eggs from commercial hens. In conclusion, some interesting nutritional features, in a preliminary way, have been highlighted that could lead the consumer to prefer these “niche” products over those obtained from commercial hybrids.

## 1. Introduction

Since the mid-twentieth century, changes in economic and social conditions went hand in hand with the growing food demand from populations, leading to the need to increase the supply of products of animal origin. In the livestock sector, a selection process of high-performing hybrid animals has been therefore undertaken, capable of significantly improving production indices, especially from a quantitative point of view. This event, had direct effects on the entire sphere of animal production, leading to the reduction of local races and breeds and consequently to an impoverishment of genetic variability for both ruminants and monogastrics [1]. In parallel with these changes, the human nutrition, generally based on a wide variety of animal and plant products, gradually moved to diets mostly composed of processed foods, therefore comprising a reduced number of species [2].

However, in the past decade a growing interest has developed in the recovery of animal breeds of zootechnical interest in danger of extinction. This phenomenon is particularly evident in developed countries, where the protection of indigenous animal breeds used for livestock production represent an important opportunity for the income diversification in disadvantaged geographic areas, therefore contributing to the maintenance of the integrity of social and economic fabric. These areas are in fact located in distant positions from the main urban centers and tend to undergo a process of marginalization that manifests itself above all through the quantitative and qualitative reduction of the local offer of public services, with consequent population decline below the critical threshold, and deterioration of both the artistic and landscape heritage [3].

The genetic erosion also characterized the poultry sector, leading to the development of synthetic hybrids able to produce higher amount of poultry meat and eggs, in comparison to the local breeds. Poultry breeding protocols, are currently focused on specialized production lines obtained through intense selection, and characterized by great genetic uniformity of the productive and qualitative traits in commercial flocks of both broilers and layers [4].

In Italy, the issue concerning the biodiversity loss in the poultry sector was particularly felt. In a study conducted by Zanon and Sabbioni [5] 90 rural poultry breeds (53 chickens, 12 turkeys, 11 guinea fowls, 9 ducks, and 5 gooses) have been specifically registered, and it is particularly indicative that 60% of these breeds can be considered already extinct, with about 13% as endangered, while only a negligible number of breeds are involved in conservation programmes. As reported by Özdemir et al. [6], in the north of the country, specifically in the Veneto region, this has led to the implementation of initiatives aimed at the diffusion of breeds in local productive systems, at the monitoring of the inbreeding process, and at the increase of knowledge on biological functions and typical morphological characteristics.

**Figure 1:**
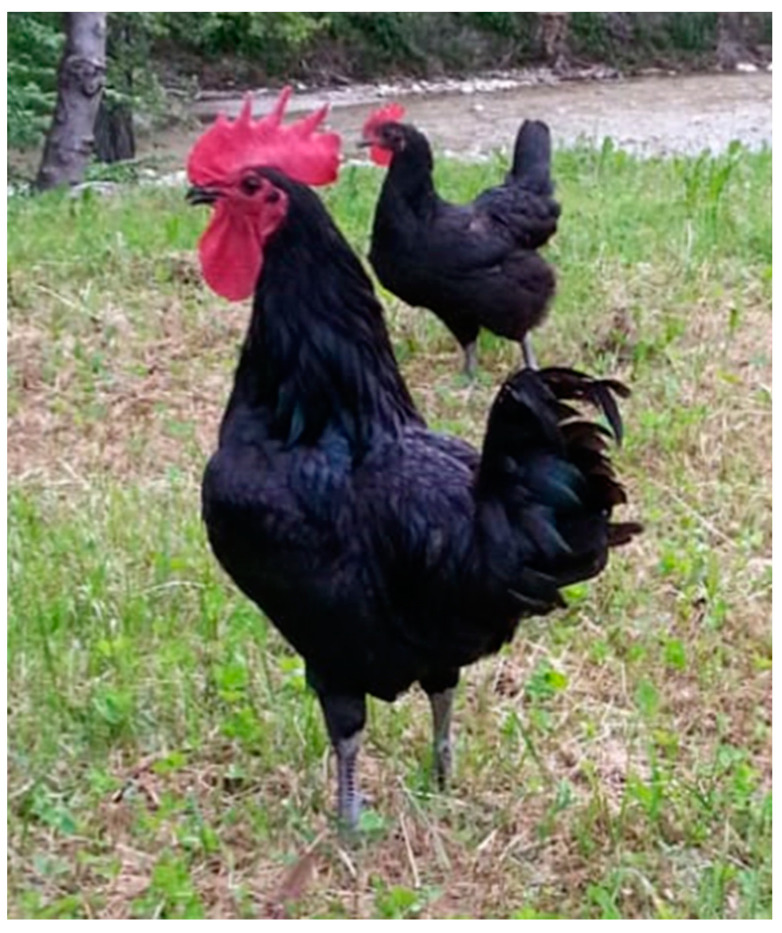
The Nera Atriana laying hen. These are hens with relatively small size, dark plumage with green, blue, and purple reflections, black skin and grey beak.

In other Italian regions there are different poultry breeds in danger of extinction that should be preserved. An example concerns the Nera Atriana laying hen, which is present in a few dozen specimens in limited areas of the Abruzzo region, and takes its name from the locality of Atri, where these animals have their greatest concentration. Few farmers are dedicated to the breeding of these animals, and their effort in preserving this poultry breed is actually not associated to commercial purposes but to reasons mostly linked to the popular tradition. These are hens of small size, able to reach a body weight of approximately 1.5–1.8 kg in adulthood, and are characterized by relatively large breasts, the plumage presents a dark-toned monocolor livery with green, blue, and purple reflections, the iris and the skin are black, while the beak is predominantly gray (Figure 1). With regard instead to the annual eggs production, given the limited number of animals and the heterogeneity in farming practices, there are currently no reliable data that can be taken into consideration and used as a reference.

Currently, no detailed characterization of the nutritional profile of egg production exists that can somehow highlight the peculiar aspects associated with this indigenous hen; hence, a study related to this is therefore necessary. For such reason this study aims to propose a first characterization of the qualitative aspects of eggs obtained from the Nera Atriana hen (AH), making a direct comparison with the chemical and nutritional characteristics of eggs obtained from a commercial hybrid reared in the same environment and applying the same feeding protocol. It is assumed that this research activity may represent an important element in the valorization of the AH in the Italian poultry sector.

## 2. Materials and Methods

### 2.1. Experimental Design, Feeding, and Sampling

The study was conducted in a commercial poultry farm located in the Abruzzo region (Italy), and all the procedures concerning the animals’ management were carried out in accordance with the Italian legislation (D.Lgs. 267/2003 of the Italian Parliament), implementing the European directives 1999/74/EC and 2002/4/EC for the protection of laying hens and the registration of the relative breeding establishments [7,8,9]. During the trial, no breeding practices other than those normally adopted were introduced, therefore approval by the ethics committee was not considered necessary.

Twenty AH and an equivalent number of ISA Brown laying hens (commercial hybrid hens-HH) with starting age of 24–25 weeks were used in this study. The study was conducted for a period of 5 months (from March to July 2019), in which animals were in the same area in which the same rearing protocol was applied. For each group of animals was created a recovery area characterized by the presence of straw on the ground, with nests for eggs deposition, and possibility of accessing an outdoor area for grazing. The animals were then raised through a traditional non-intensive practice with a density greater than 4 m^2^/hen; furthermore animals had access to outdoor spaces for the most of the day avoiding the need of applying artificial light. Water was provided ad libitum, and all animals received the same diet for laying hens in production whose composition, chemical characterization, and fatty acid profile are reported in Table 1. Feed was analyzed for dry matter (DM), crude protein, lipid, crude fiber, ash, calcium, phosphorus, and sodium according to the Commission Regulation (EC) N. 152/2009 [10] in which are reported the official procedures for sampling and analytical evaluations on feeds.

At the start of the trial and every 4 weeks, 15 eggs from each groups of hens were collected for physical and chemical-nutritional evaluations; specifically, reference was made to 6 collection time points for a total of 90 eggs sampled per group of hens over the course of the trial. The albumen, yolk, and shell weights were immediately analyzed within 48 h from collection, as well as the yolk color and albumen pH; while the evaluations concerning the determination of β-carotene, the characterization of the fatty acid profile, the cholesterol dosage and the characterization of the yolk protein profile were performed on yolk aliquots stored at −20 °C until analysis.

### 2.2. Physical Analysis and Yolk Color

The weight of whole eggs and their main components (shell, albumen and yolk) was determined by using an electronic balance (ENTRIS124-1S; d = 0.1 mg; Sartorius Lab Instruments GmbH & Co., Goettingen, Germany); this made it possible to attribute a relative percentage to each egg component. Subsequently, the pH of the albumen was calculated by using a pH meter equipped with an electrode (Crison, Barcelona, Spain) that was thoroughly rinsed with distilled water after each measurement. Standard phosphate buffers (pH 4.00 and 7.00) were used to calibrate the pH meter before the analysis.

The egg yolk color was evaluated by using a Bench-top Colorimeter CR-5 Konica Minolta (Minolta corp., Ramsey, NJ, USA), by following the procedure previously described by Ianni et al. [11]. Briefly, reference was made to the Commission Internationale de L’Eclairage (CIE) L*a*b* system, in which the color space can be defined in terms of lightness (L*), redness (a*) and yellowness (b*). Specifically, L* is expressed in percentage units (0 for black and 100 for white), while values ranging from −60 to +60 are used to represent a* and b*. The whole yolks were homogeneously distributed into a glass petri dish and the color was recorded in five different part. The measurements were performed in reflectance and the instrument was equipped with an automatic white calibration. For each evaluation, the chroma-meter was set perpendicular to the sample surface.

### 2.3. Determination of β-carotene in Feed and Egg Yolks

The β-carotene determination in feed and yolk samples was performed according to the AOAC method 43.015 [12] with slight modifications. One gram of sample was mixed with 10 mL of acetone and left for 40 min in the dark at 20–22 °C (room temperature). After filtering the solution, was added further acetone in order to reach a final volume of 20 mL. One and a half mL of the obtained extracts were then used for the spectrophotometric evaluation at 450 nm by using an UV-VIS spectrophotometer (Jenway, Essex, UK). The β-carotene quantification was obtained by preparing a calibration curve with purified β-carotene (Sigma-Aldrich, Milan, Italy). The curve was linear in the range of concentration between 1 and 100 μg/mL (R^2^ = 0.9895).

### 2.4. Analysis of the Fatty Acids Profile in Feeds and Egg Yolks

The total lipid dosage, both in feed and in the egg yolks, was performed by following the procedure previously described by Innosa et al. [13].

With regard to the characterization of the fatty acid composition, about 1.5 g of each sample were weighed and homogenized in 20 mL of a solution containing chloroform and methanol (2:1 *v/v*). Then, samples were kept in continuous agitation for 3 h and subsequently transferred in separating funnels in a sodium chloride solution. After 15 h in the dark, the separation of the chloroform phase took place, which was recovered. Total fat in each sample was obtained by removing the solvent with a strike-rotating evaporator (Steroglass s.r.l., Perugia, Italy). Samples were then recovered with 1 mL of hexane and transferred into 5-mL vials for the esterification reaction in presence of 500 μL of MeONa. The detection of the fatty acid methyl esters (FAME) was performed as previously described [14], by using a gas chromatograph (Focus GC; Thermo Scientific, Waltham, MA, USA) equipped with a capillary column (Restek Rt-2560 Column fused silica 100 m 0.25 mm highly polar phase; Restek Corporation, Bellefonte, PA, USA) and a flame ionization detector (FID). The temperature program envisaged an initial step of 1 min at 175 °C, followed by a temperature increase to the final temperature of 215 °C at a rate of 2 °C/min, a condition that was maintained for 50 min until the end of the chromatographic run. The area of obtained peaks was quantified by exploiting the Chrome-Card software, and the values associated with individual fatty acids (FA) were expressed as relative percentage of total detected FAME. The retention times recorded with the standard mixture FIM-FAME7-Mix (Matreya LLC, State College, PA, USA) were helpful in the identification of individual FAME. The value attributed to each FA was used to calculate the sum of total saturated fatty acid (SFA), monounsaturated fatty acid (MUFA), and polyunsaturated fatty acid (PUFA). In addition to this, the desaturation indices (DI) were associated to palmitic (C16:0) and stearic (C18:0) acids, according to the formulas applied by Brogna et al. [15]. 

### 2.5. Cholesterol Quantification in Egg Yolks

The cholesterol extraction from egg yolks has been performed according to the procedure previously described by Innosa et al. [13] with slight modifications. For each sample, about 10 g of egg yolk was saponified by using 10 mL of ethanol, 500 μL of butylated hydroxytoluene (BHT) 20% in methanol solution, and 100 μL of KOH 50% in water solution. Following homogenization, samples were placed for 30 min in a water bath at 60 °C. After cooling, 8 mL of ether petrolatum, 1 g of NaCl and 2 mL of distilled water were added to each sample. Then, the samples were sonicated and centrifugated in order to obtain a supernatant containing non-saponifiable lipids as cholesterol. A strike-rotating evaporator (Steroglass s.r.l., Perugia Italy) was used to remove the solvent and non-saponifiable lipids were recovered with 1 mL of hexane and transferred into an 8-mL vial. At this point, samples were derivatized by using pyridine and N,O-bis-(trimethylsilyl)-trifluoroacetamide (BSTFA) in a 2:1 ratio.

The analysis was performed with a gas chromatography coupled with a mass spectrometry (GC-MS; Perkin Elmer, Walthman, MA). The instrument was equipped with an Elite-5ms column (length: 30 m; internal diameter: 0.25 mm; film thickness: 0.25 μm; Perkin Elmer, Waltham, MA, USA) and helium was exploited as carrier gas with a flow rate of 1 mL/min. The temperature program of the oven was initially set at 180 °C and held for 1 min, then increased up to 250 °C with progression of 2 °C/min and immediately increased to the final temperature of 300 °C with a ratio of 5 °C/min and held for 6 min. The analytical method for the cholesterol identification was calibrated in the range from 1 to 50 mg/mL (R^2^ = 0.996040).

### 2.6. Yolk Proteins Evaluation with SDS-PAGE

The yolk protein profile was evaluated under reducing conditions through the sodium dodecyl sulfate polyacrylamide gel electrophoresis (SDS-PAGE) following the procedure reported by Guilmineau et al. [16] with slight modifications. Specifically, in each lane volumes of sample corresponding to a total protein amount of 10 µg were loaded and the electrophoretic run was performed in 12% polyacrylamide gels at 150 V for 120 min. The ImageJ software was used for the densitometric analysis of the visualized bands [17].

### 2.7. Statistical Analysis

The statistical analysis was performed by using SigmaPlot 12.0 Software (Systat software Inc., San Jose, CA, USA) for windows operating system. The one-way ANOVA model was applied, and the post-hoc comparison was performed through the Tukey’s test; *p* values lower than 0.05 were considered statistically significant.

## 3. Results

### 3.1. Characterization of the Basic Physicochemical Properties

In Table 2 are reported all the data relating to the physical parameters evaluated on eggs sampled during the experimental period. The analysis of the average weight of whole eggs showed significantly higher values for eggs obtained from commercial hybrid laying hens (59.84 ± 5.92 g vs. 44.55 ± 2.59 g for HH and AH respectively, *p* < 0.01). The same evidence was also found for albumen (*p* < 0.01) and yolk (*p* < 0.05) belonging to the HH samples while, in the case of the shell, no significant differences were evidenced, although a higher mean value was noted for HH samples with a *p* value equal to 0.0511. Taking into consideration the percentage composition of the single components, the data concerning the egg albumen were confirmed (63.40 ± 5.23% vs. 56.48 ± 5.81% in HH and AH respectively, *p* < 0.05), while in the case of yolk and shell the percentages found in the AH eggs were significantly higher (*p* < 0.05 for both).

In Table 2 are also reported the data concerning the evaluations of the egg yolks color. The analysis evidenced no variations with regard to the lightness (L*) and yellowness (b*), while the chromatic coordinate a* (redness) resulted significantly higher in AH samples (*p* < 0.01).

The yolks were also subjected to analysis of the basic chemical-nutritional profile, with specific reference to the dosage of total lipids, β-carotene, and total cholesterol (Table 3). In all three cases no significant differences were observed (*p* > 0.05), although it should be reported that the *p* value associated with cholesterol showed a trend toward significance as it was lower than 0.1 (*p* = 0.0521).

### 3.2. Fatty Acid Composition in Egg Yolks

The characterization of the fatty acid profile in HH and AH yolks highlighted specific differences that are shown in Table 4. Specifically, the yolk obtained from the indigenous laying hens was characterized by a higher concentration of palmitic acid (C16: 0; *p* < 0.01) resulting in an overall higher relative percentage of total saturated fatty acids in AH samples (*p* < 0.01). With regard to MUFA, the AH samples showed a higher relative percentage of palmitic acid (C16:1 cis-9; *p* < 0.05) and a significantly lower value associated with oleic acid (C18:1 cis-9; *p* < 0.05). The only PUFA found in the yolk samples were linoleic (C18:2 cis-9, cis-12), linolenic (C18:3 cis-9, cis-12, cis-15), and arachidonic (C20:4 cis-5, cis-8, cis-11, cis-14) acids without significant differences between egg yolks obtained from commercial hybrid and Nera Atriana laying hens. The differences observed at the level of the single fatty acids resulted effective in inducing an overall significantly higher relative percentage of total SFA in the AH samples (*p* < 0.01) and no difference as regards total MUFA and PUFA (*p* > 0.05). Even the DI, calculated for C16:1 and C18: 1, did not show any noteworthy variation (*p* > 0.05).

### 3.3. Protein Profile Characterization under Reducing Conditions

**Figure 2:**
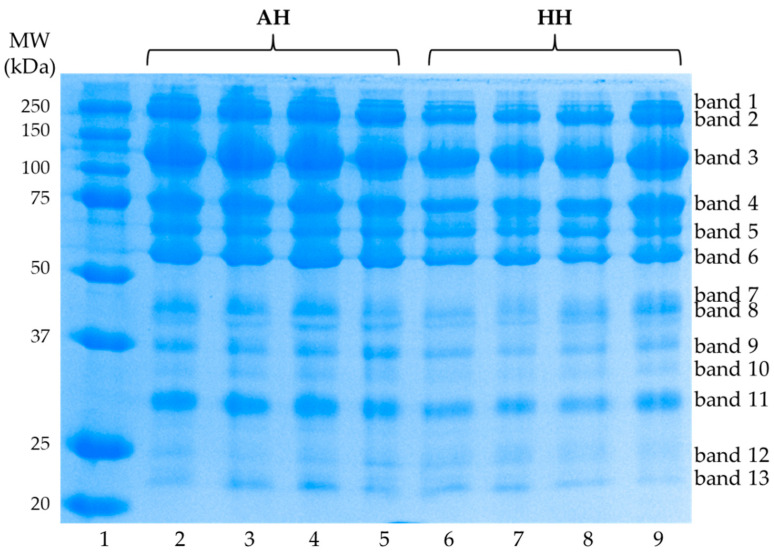
Sodium dodecyl sulfate polyacrylamide gel electrophoresis (SDS-PAGE) of globular proteins extracted from egg yolks obtained from Nera Atriana laying hens (AH-lanes from 2 to 5) and commercial hybrid hens (HH-lanes from 6 to 9). In lane 1 the molecular weight (MW) markers were loaded. The figure is representative of all the analyzed samples.

The SDS-PAGE performed under reducing conditions allowed to identify in all the analyzed samples 13 electrophoretic bands with molecular weight (MW) ranging from 250 to 22 kDa (Figure 2).

In Table 5 are reported the results of the densitometric analysis of the visualized bands. The only significant variations have been evidenced for the protein fractions corresponding to bands 10 (33.33 kDa), 11 (29.86 kDa), and 12 (24.79 kDa). Bands 10 and 11 showed a higher intensity in the AH samples (*p* < 0.05 for band 10 and *p* < 0.01 for band 11, respectively) while for band 12 a significantly higher density has been found in samples obtained from the commercial hybrid hen (*p* < 0.01).

## 4. Discussion

In this study the main physical and chemical properties of the eggs obtained from Italian indigenous laying hens, reared in limited areas of central Italy have been characterized. This approach had as its main purpose the attempt to compare these parameters with those concerning commercial eggs, in order to highlight some useful characteristics to valorize a genetic resource that is highly at risk of extinction.

The evaluation of the total egg weight showed a significantly higher value for eggs obtained from ISA Brown hens, a finding in full agreement with what previously reported by other authors who performed a direct comparison between indigenous and commercial hybrid breeds. Pintea et al. [18] found that Araucana hens, a local breed, produced significantly smaller eggs than those obtained by ISA Warren hens. A similar observation was also reported by Sokołowicz et al. [19] who presented a study in which was evaluated the quality of organic eggs from hens of different genotypes and ages, and by Lukanov et al. [20] who compared the egg quality characteristics between Araucana and Schijndelaar with highly productive White Leghorn and Rhode Island Red strains. Obviously, it must be also said that these evidences confirm a fairly expected data, taking into account that commercial strains have been suitably selected for the high egg productions.

In addition to what has been reported, in eggs obtained from ISA Brown hens the albumen relative percentage resulted to be higher while the yolk percentage lower than those observed in eggs from Nera Atriana hen. Also this data confirmed a previously characterized phenomenon, that was already observed in the comparison between commercial hybrid and local breeds. Moreover those studies in which the finding has been evidenced and confirmed by conducting evaluations on Italian breeds should be mentioned [21,22].

A finding of particular interest regards the data of the relative percentage of the shell, which is higher in the eggs obtained from the Nera Atriana hen than in those produced by the commercial hybrid breed. Previous studies in which the same phenomenon was found, have highlighted the correlation between this result with advantages above all from an economic point of view, but also with regard to the issue of consumer safety [23,24]. It is in fact well-known that the eggs that break during the production and handling processes, represent an economic damage for the operators of the sector. For this reason, having a product more resistant to physical stress is certainly not a negligible aspect. In addition to this it should be emphasized that in many cases, the presence of even minimal lesions on the shell structure can expose the product to be colonized by microbial agents potentially harmful for the consumers’ health. These issues have been extensively characterized in the past [24,25] and it is important to underline that also other studies focused on the qualitative aspects of eggs obtained from local and not very widespread poultry breeds, have shown similar findings in comparison with eggs produced by commercial hybrid breeds [22].

In the case of the yolk color, reference is made to a parameter which is not always well correlated with nutritional determinations. In most cases this is a characteristic associated with the degree of acceptability of the consumer, who tends to prefer eggs that have a more intense yolk color. In this study no variations have been observed for yolk lightness (L*) and yellowness (b*), while higher redness (a*) was evidenced in AH egg yolks. In study previously conducted by Hammershøj et al. [26], the chromatic coordinate a* was reported to correlate with the β-carotene concentration in the diet; however in our study no differences were introduced in the diet and, as reported above, no differences in concentration of β-carotene in the yolk were found, thus dispelling any doubts about any differences in the degree of absorption of this compound by the hens. For this reason it is presumed that the difference may have been determined by other compounds present in the diet, such as lutein for instance, which has shown the ability to influence this parameter in other previous studies [27].

β-carotene is a bioactive compound to which are attributed interesting health properties, specifically related to its marked radical-scavenging antioxidant potential [28]. The presence of this compound in food products has been associated with significant advantages regarding the product conservation and benefits for consumers health [29,30]. For this reason, there is growing interest in the development of protocols useful for the production of functional foods enriched with this compound. However, as recently discussed by Spasevski et al. [31], in food enrichment processes a “synthetic” β-carotene which lacks the 9-cis isomer is generally used. This isomer is instead present in natural matrices and is characterized by greater liposolubility and antioxidant activity in comparison to the other isomers, as well as greater resistance to degradation processes that could be induced for instance by heating treatments [32]. For this reason, the strategy of obtaining animal products naturally enriched with this compound is widely shared, by administering to farm animals a diet richer in this compound, or through the selection of animals with a metabolism capable of guaranteeing a more efficient absorption of the compound from feed [33,34]. In our study, all animals received the same diet for the entire experimental period, therefore the evaluation of the concentration of β-carotene in the yolk was aimed at determining the ability of the two poultry breeds to absorb the compound from the diet and transfer it to the eggs. From this point of view, no significant differences were highlighted, it is however interesting to be able to state that from this point of view the metabolism of the Nera Atriana hen is similar to that of a commercial hybrid breed.

Between the HH and AH yolk samples no differences in the content of total lipids and cholesterol were highlighted. However, in the case of cholesterol, a tendentially lower average value must be highlighted in the eggs obtained from the Nera Atriana hen, with a *p* value just above the limit of statistical significance (*p* = 0.0521). These data are obviously very interesting and deserve to be further investigated, possibly by monitoring the data over longer time intervals and on an even greater number of samples, or examining the cholesterol level at different time points, as there are numerous studies that show that yolk cholesterol content is hen-age dependent. The presence of limited concentrations of cholesterol in food is a factor of considerable interest, above all due to the potentially negative effects on consumer health. A frequent consumption of foods rich in cholesterol is in fact associated with chronic diseases, especially atherosclerosis; furthermore, in the presence of certain conditions, cholesterol tends to oxidize, giving rise to compounds, the oxysterols, that are also capable of inducing negative effects at the cardiovascular level [13,35]. Over time, food technology has tried to develop strategies aimed at obtaining food products low in cholesterol [36]; in the zootechnical field a lot has been done trying to vary the diet of the animals, but obviously the advantage is undisputed in the case in which the chemical-nutritional properties of a food product are essentially the result of the animal genetics.

The genetic factor showed instead the ability to significantly affect the fatty acids profile in eggs. The first noteworthy difference concerns the greater presence of palmitic acid (C16:0) in the AH samples. Since the diet administered to the animals was the same, these data should be totally attributed to a different endogenous synthesis, with the probable involvement of the fatty acid synthase (FAS). The animal FAS is a multifunctional enzyme with a molecular weight of about 260 kDa, in which the catalytic sites are arranged as a series of connected globular domains. The enzymatic reactions mediated by FAS are essentially the same in all organisms and are collectively responsible for the de novo production of palmitate which is synthesized from acetyl-coA, malonyl-CoA, and NADPH [37]. From this point of view, to date there are no specific studies on the expression and activity of this enzyme in Nera Atriana hen, however it could be attempted to discuss this data by exploiting the information previously collected in experimentations on pigs. Specifically such studies showed the tendency of indigenous pig breeds, in contrast to hybrid breeds, to accumulate higher amount of intramuscular lipids as a consequence of the increased expression of transcription factors and genes associated to the fatty acid metabolism in different tissues [38,39].

The presence in HA samples of higher quantities of palmitoleic acid (C16: 1 cis-9) represents a strict consequence of the higher concentration of C16:0 in the same samples. These data are confirmed by the presence of similar desaturation indices, testifying to an almost identical activity of the Δ^9^-desaturase, which simply has more substrate at its disposal in the case of the Nera Atriana hen. Also the desaturation index of oleic acid (C18:1 cis-9) did not show differences from a statistical point of view, therefore the greater concentration of this compound should be directly related to the amount of stearic acid (C18:0) produced. The concentration of C18:0 was in fact slightly higher in the HH samples, although this difference was not significant.

Overall, was also evidenced a higher content of saturated fatty acids in eggs produced by the Nera Atriana hen. Commonly this aspect is not particularly positive as an excess of these compounds involves greater risks for the consumers’ health. On the other hand, these are also compounds less susceptible to oxidation if exposed to stress of various origin, both physical and chemical [13]. Since eggs tend to be consumed preferably cooked, it can be assumed that AH eggs could be more resistant to thermal stress, avoiding the formation of secondary volatile compounds, especially aldehydes, ketones, and alcohols, capable of negatively influencing both the taste and the product shelf-life [40].

Regarding the analysis of the protein component in yolk, it is necessary to consider the fact that most of the yolk proteins are associated with the lipid component to form lipoproteins that are generally classified in high-density lipoproteins (HDL) and low-density lipoproteins (LDL). However, a small percentage of lipid-free globular glycoproteins consisting of water soluble structures corresponding to the blood serum proteins of the animal is also present. In this study the electrophoretic approach under reducing conditions proved very useful in the analysis of the granular protein fraction of the yolk, and in all the analyzed samples has been highlighted an electrophoretic profile comparable to what has been previously reported by other authors [16,41].

The only differences evidenced in the analyzed samples concerned three low molecular weight protein fractions (33.33 kDa, 29.86 kDa, and 24.79 kDa, respectively). Among these, the condition of greatest interest concerns the protein fraction with the highest molecular weight, as it should correspond to β-livetin. The identification has been performed by taking into account the electrophoretic profile previously characterized by Guilmineau et al. [16] under reducing conditions, although other authors indicate a slightly higher reference molecular weight for this protein, corresponding approximately to 45 kDa [42]. β-livetin has been identified as an α-2-glycoprotein, to which has been attributed an important anti-inflammatory function. Specifically, an in vitro study conducted by Meram and Wu [43] on lipopolysaccharide (LPS)-induced RAW 264.7 macrophages, recently demonstrated the ability of α-, β-, and γ-livetin to inhibit the nitric oxide (NO) production as a consequence of a reduced expression of the inducible nitric oxide synthase (iNOS); furthermore a significant decrease in the release of pro-inflammatory cytokines, such as interleukin-1β (IL-1β), interleukin-6 (IL-6), and tumor necrosis factor-α (TNF-α) was observed. In the analysis conducted by Guilmineau et al. [16], an evaluation of the thermal sensitivity of egg yolk proteins was also performed, finding the tendency of β-livetin to resist the denaturation process induced by heating at 74 °C for 15 min. By transferring this finding to the food sector, it could mean greater resistance to the thermal stress induced by the cooking processes, allowing this functional compound to maintain its structural integrity at the time of consumption. However, this aspect should be better characterized since other authors report conflicting data on the thermal sensitivity of β-livetin [44,45,46].

## 5. Conclusions

The characterization of the qualitative parameters of eggs obtained from the Nera Atriana hen, has highlighted some aspects that could be useful for the valorization of the animal product. In comparison with commercial hybrid hens, Nera Atriana hen eggs were characterized by a higher relative percentage of both the shell and the yolk, and a more intense yolk redness, with presumable positive implications on the product integrity and consumer acceptability. Furthermore, a composition in fatty acids was observed which would justify a lower susceptibility to lipoperoxidation processes, the presence of protein fractions credited of potential benefits for human health, and a lower tendency of eggs yolk to accumulate cholesterol, although this last datum needs to be better characterized. This study should therefore be understood as a preliminary approach aimed at ensuring that interest can develop in preserving a genetic resource currently at risk of extinction.

## Figures and Tables

**Table 1:** Chemical composition and fatty acid profile of the diet administered to the animals involved in the trial.

Ingredients of the Diet (%)	
Maize	25
Decorticated sunflower	17
Common wheat	13
Field beans	11
Sorghum	10
Calcium carbonate	9.5
Barley	7.5
Bran	6
Dicalcium phosphate	0.5
Vitamins	0.5
**Chemical Composition of the Diet (%)**	
Dry matter	88.20
Crude Protein ^1^	15.40
Ether extract ^1^	2.70
Fiber ^1^	5.10
Ash ^1^	12.20
Calcium ^1^	3.90
Phosphorus ^1^	0.49
Sodium ^1^	0.20
**β-carotene (µg/g)**	25.98 ± 1.62
**Fatty Acid Profile (%) ^2^**	
C16:0	11.23 ± 0.88
C18:0	3.82 ± 0.27
C18:1 cis-9	24.95 ± 2.05
C18:2 cis-9, cis-12	55.76 ± 3.73
C18:3 cis-9, cis-12, cis-15	4.24 ± 0.38

^1^ Values are reported on a dry matter (DM) basis. Data concerning β-carotene and fatty acid profile are reported as mean values ± S.D. ^2^ Data are reported as mean relative percentages of total FAMEs. Analysis concerning β-carotene and FA composition were performed in triplicate on 3 different samplings.

**Table 2:** Physical properties of eggs obtained from commercial hybrid hen (HH) and Nera Atriana laying hen (AH).

Trait ^1^		HH	AH	*p* Value
Egg weight, (g)		59.84 ± 5.92	44.55 ± 2.59	**
Albumen weight, (g)		37.94 ± 3.13	25.16 ± 2.43	**
Yolk weight, (g)		14.66 ± 0.92	12.96 ± 0.86	*
Shell weight, (g)		7.24 ± 0.81	6.43 ± 0.86	ns
Albumen, (%)		63.40 ± 5.23	56.48 ± 5.81	*
Yolk, (%)		24.50 ± 1.54	29.09 ± 1.93	*
Shell, (%)		12.10 ± 1.35	14.43 ± 1.93	*
Albumen pH		8.62 ± 0.02	8.64 ± 0.03	ns
Yolk color	L *	34.61 ± 3.26	34.62 ± 4.66	ns
	a *	−6.31 ± 0.57	−3.32 ± 0.41	**
	b *	44.14 ± 4.75	42.65 ± 4.08	ns

^1^ Data are reported as mean values ± S.D. (n = 90); * *p* < 0.05; ** *p* < 0.01; ns: not significant.

**Table 3:** Total lipids, β-carotene, and cholesterol content in egg yolks obtained from commercial hybrid hen (HH) and Nera Atriana laying hen (AH).

Trait ^1^	HH	AH	*p* Value
Total lipids, (%)	23.85 ± 2.28	25.04 ± 3.55	ns
β-carotene, (µg/g)	19.14 ± 1.71	17.98 ± 1.66	ns
Cholesterol, (mg/g)	12.74 ± 0.93	12.26 ± 0.81	ns

^1^ Data are reported as mean values ± S.D. (n = 90); ns: not significant.

**Table 4:** Fatty acid profile in egg yolks obtained from commercial hybrid hen (HH) and Nera Atriana laying hen (AH).

Fatty Acids ^1^	HH	AH	*p* Value
C14:0	0.43 ± 0.05	0.45 ± 0.06	ns
C16:0	24.79 ± 2.06	28.58 ± 2.51	**
C18:0	8.49 ± 0.87	9.46 ± 1.07	ns
SFA	33.71 ± 3.08	38.49 ± 3.27	**
C16:1 cis-9	1.92 ± 0.23	2.94 ± 0.28	*
C18:1 cis-9	44.53 ± 3.83	39.31 ± 3.35	*
MUFA	46.45 ± 4.01	42.25 ± 3.71	ns
C18:2 cis-9, cis-12	14.34 ± 1.32	14.13 ± 1.40	ns
C18:3 cis-9, cis-12, cis-15	0.38 ± 0.05	0.30 ± 0.04	ns
C20:4 cis-5, cis-8, cis-11, cis-14	2.25 ± 0.19	2.12 ± 0.21	ns
PUFA	16.97 ± 1.55	16.55 ± 1.63	ns
Others	2.87 ± 0.29	2.69 ± 0.26	ns
DI (C16:1 cis-9)	0.07 ± 0.01	0.09 ± 0.11	ns
DI (C18:1 cis-9)	0.84 ± 0.10	0.81 ± 0.09	ns

^1^ Data are reported as mean relative percentages of total FAMEs ± S.D. (n = 90); SFA: saturated fatty acids; MUFA: monounsaturated fatty acids; PUFA: polyunsaturated fatty acids; DI: desaturation index; DI (C16:1 cis-9) has been calculated as follow: [C16:1 cis-9/C16:1 cis-9 + C16:0)]; DI (C18:1 cis-9) has been calculated as follow: [C18:1 cis-9/C18:1 cis-9 + C18:0)]; * *p* < 0.05; ** *p* < 0.01; ns: not significant.

**Table 5:** Densitometric analysis of electrophoretic bands visualized through the sodium dodecyl sulfate polyacrylamide gel electrophoresis (SDS-PAGE) of globular proteins extracted from egg yolks obtained from commercial hybrid hen (HH) and Nera Atriana laying hen (AH).

Band No. ^1^	MW ^2^ (kDa)	AH ^3^	HH ^3^	*p* Value
1	250.00	2.57 ± 0.56	3.18 ± 0.72	ns
2	233.07	10.81 ± 1.23	10.81 ± 0.92	ns
3	116.70	25.85 ± 2.08	25.16 ± 3.48	ns
4	74.36	12.15 ± 1.17	12.45 ± 1.49	ns
5	64.35	6.22 ± 1.95	8.07 ± 2.29	ns
6	55.37	11.63 ± 0.83	11.04 ± 1.22	ns
7	43.32	6.42 ± 0.80	6.35 ± 1.91	ns
8	41.00	1.58 ± 0.62	1.26 ± 0.31	ns
9	36.50	3.62 ± 1.03	2.95 ± 0.54	ns
10	33.33	1.34 ± 0.20	1.07 ± 0.26	*
11	29.86	12.61 ± 0.88	10.67 ± 0.99	**
12	24.79	1.54 ± 0.45	3.85 ± 1.26	**
13	22.18	3.65 ± 0.97	3.14 ± 1.09	ns

^1^ Reference is made to the numbering reported in Figure 2; ^2^ The molecular weight (MW) has been calculated by exploiting the markers used in the SDS-PAGE (Figure 2); ^3^ Data are reported as mean percentages ± S.D. (n = 30); * *p* < 0.05, ** *p* < 0.01, ns: not significant.

## Data Availability

The data presented in this study are available on request from the corresponding author.
